# Psychopharmacological treatment in elderly patients

**DOI:** 10.1192/j.eurpsy.2025.249

**Published:** 2025-08-26

**Authors:** M. Siwek

**Affiliations:** Department of Affective Disorders, Jagiellonian University Medical College, Krakow, Poland

## Abstract

**Abstract:**

Mental disorders in the elderly occur much more often than in young adults, and their prevalence increases significantly with age and in the case of people receiving institutional care. Psychopharmacotherapy in an elderly patient is much more challenging and requires more restrictive safety strategies than in younger patients. The significantly increased risk of interactions, side effects and complications resulting from: comorbidities, systemic changes due to aging and, as well as their pharmacotherapy, are a serious problem in the treatment of elderly patients. It should be emphasized that the safety profile may be different in the elderly compared to the young adults - some complications that are rare in younger patients, e.g. bleeding complications, fractures and bone loss, cataract progression, hyponatremia, falls, QT prolongation, stroke, pneumonia and others, are a much more common problem among seniors and their risk should be considered at the initial stage of pharmacotherapy selection. In the case of some drugs (e.g. antipsychotics), the accumulation of serious complications may significantly increase the risk of premature mortality, if they are used incorrectly. Moreover, it should be remembered that certain side effects, e.g. orthostatic hypotension, may occur with drugs for which these side effects are unlikely in younger patients. During the treatment process, it is also necessary to monitor the presence and severity of side effects, such as: constipation, dry mouth, tremors, urination problems, excessive sleepiness or cognitive impairment, which may reduce the quality of life, particularly in an elderly patient. This presentation will provide a brief and practical clinical guide to safe and effective pharmacotherapy in the elderly patients.

**Disclosure of Interest:**

None Declared

